# The Shifting Paradigm of Monoclonal Antibodies in COVID-19 Management: From Early Triumphs to Viral Resistance and Future Perspectives

**DOI:** 10.3390/antib15030048

**Published:** 2026-06-11

**Authors:** Francesco Ferrara, Flavia De Berardinis, Manlio Scognamiglio, Andrea Zovi

**Affiliations:** 1 Pharmaceutical Department, ASL Napoli 3 Sud, 80035 Naples, Italy; 2 Department of Mental Health, ASL Roma 3, 00121 Rome, Italy; flavia.deberardinis@aslroma3.it; 3 Pharmaceutical Department, ASL Salerno, 84025 Salerno, Italy; mn.scognamiglio@aslsalerno.it; 4 School of Pharmacy, University of Camerino (Macerata), 62032 Macerata, Italy; zovi.andrea@gmail.com

**Keywords:** SARS-CoV-2, COVID-19, monoclonal antibodies, Omicron, immune escape, spike protein, tocilizumab, antimicrobial stewardship, hospital pharmacy

## Abstract

Background: Monoclonal antibodies (mAbs) initially played a major role in outpatient COVID-19 management by providing rapid passive immunity and reducing progression to severe disease. However, continuous SARS-CoV-2 evolution progressively compromised the effectiveness of several anti-spike products. This narrative review summarizes the trajectory of COVID-19 mAbs across three phases: early clinical efficacy, loss of efficacy due to immune escape, and future directions. Methods: We conducted a narrative review focusing on mechanisms of action, pivotal clinical trials, and real-world effectiveness of neutralizing anti-spike mAbs and host-directed immunomodulatory mAbs. Emphasis was placed on the impact of variants—especially Omicron—on susceptibility and clinical use, as well as on emerging next-generation platforms. Results: First-generation neutralizing mAbs substantially reduced the hospitalization rates during the Alpha and Delta waves, while immunomodulatory mAbs became standard options for the hyperinflammatory phase in hospitalized patients. With the emergence of Omicron and its sub-lineages, extensive immune escape led to marked reductions in neutralization for many earlier anti-spike agents and consequent restrictions in use. Later-generation approaches targeting more conserved epitopes provided temporary solutions but were also challenged by ongoing antigenic drift. Host-directed immunomodulators retained clinical relevance because their mechanism is independent of viral spike mutations. Conclusions: The clinical role of monoclonal antibodies in COVID-19 has been dynamic and increasingly constrained by viral evolution. Future strategies should prioritize broadly neutralizing antibodies targeting conserved epitopes, innovative delivery platforms, and integration with real-time surveillance to preserve clinical utility in the endemic phase and improve preparedness for future outbreaks.

## 1. Introduction

**Figure 1:**
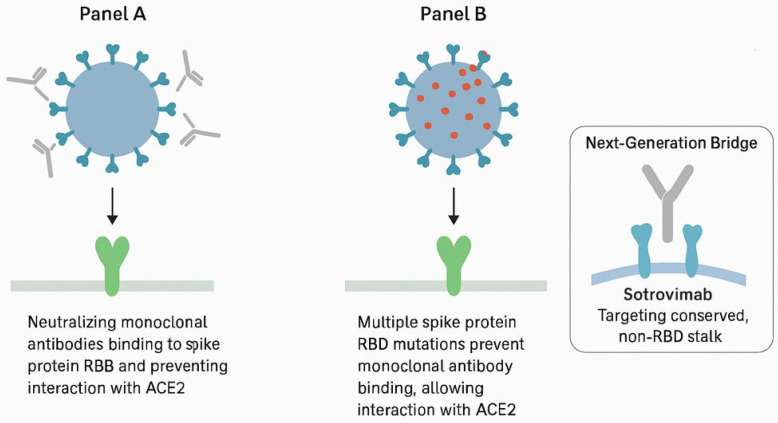
The evolutionary warfare: Omicron’s mutational escape from early monoclonal antibodies. Schematic representation of Omicron-driven immune escape from early neutralizing monoclonal antibodies. Panel A: In pre-Omicron variants, neutralizing monoclonal antibodies bind epitopes on the viral spike receptor-binding domain (RBD), thereby preventing the spike– the human angiotensin-converting enzyme 2(ACE2) interaction. Panel B: Omicron-lineage spike mutations (particularly within the RBD) reduce antibody binding, allowing for efficient interaction between spike and ACE2. ACE2 is depicted consistently across panels to avoid misinterpretation.

The emergence of Severe Acute Respiratory Syndrome Coronavirus 2 (SARS-CoV-2) in late 2019 triggered a global health crisis that profoundly challenged healthcare systems and clinical decision-making worldwide [1]. Coronavirus Disease 2019 (COVID-19) displays marked clinical heterogeneity, ranging from asymptomatic infection to acute respiratory distress syndrome (ARDS), systemic thromboinflammation, multiorgan failure, and death [2]. In the early stages of the pandemic, management was largely limited to supportive care, while the urgent need for effective targeted therapies accelerated drug-development pathways [3]. In parallel with the rapid deployment of vaccines, recombinant monoclonal antibodies (mAbs) emerged as a key pharmacological strategy to provide rapid passive immunity in high-risk individuals and to curb disease progression when administered early in infection [4,5]. Early discussions emphasized the complementary role of mass vaccination and neutralizing mAbs as coordinated tools to reduce severe outcomes in vulnerable populations [6]. However, SARS-CoV-2 has continued to evolve through successive variants of concern (VOCs), many of which display substantial immune-evasion properties that directly impact the effectiveness and sustainability of anti-spike antibody therapies [7]. This narrative review critically examines the full trajectory of mAbs in COVID-19, from early clinical success to Omicron-driven loss of activity and future perspectives, while also considering the operational and stewardship implications for healthcare systems (Figure 1).

## 2. Viral Pathogenesis, Immune Evasion, and Therapeutic Targets

To fully understand the rationale and mechanisms of monoclonal antibody (mAb) therapy, it is essential to outline key aspects of SARS-CoV-2 pathogenesis. SARS-CoV-2 is an enveloped, positive-sense, single-stranded RNA virus [8]. Its genome encodes several structural proteins, including spike, envelope, membrane, and nucleocapsid. Among these, the spike (S) glycoprotein represents the most clinically relevant target for neutralizing mAb development [9]. The S protein is a trimeric class I fusion glycoprotein composed of two functional subunits: S1, which contains the highly variable receptor-binding domain (RBD), and S2, which is comparatively conserved and mediates membrane fusion [10]. Viral entry occurs through a two-step process. First, the S1 RBD binds with high affinity to the human angiotensin-converting enzyme 2 (ACE2) receptor, which is expressed on alveolar epithelial cells, vascular endothelium, and enterocytes [11]. Second, host proteases—most notably transmembrane serine protease 2 (TMPRSS2) and furin—cleave and activate the S protein, exposing the fusion peptide within S2 and enabling fusion between the viral envelope and the host cell membrane [12]. Neutralizing mAbs are engineered to bind specific epitopes on the RBD, thereby sterically hindering the spike–ACE2 interaction and neutralizing free virions [13]. Beyond direct neutralization, Fc-mediated effector functions may contribute to in vivo activity. By engaging Fc gamma receptors (FcγRs) on innate immune cells (e.g., natural killer cells and macrophages), IgG-based mAbs can promote antibody-dependent cellular cytotoxicity (ADCC), antibody-dependent cellular phagocytosis (ADCP), and complement-dependent cytotoxicity (CDC), facilitating the clearance of infected cells [14]. In severe COVID-19, the dominant pathophysiology may shift from active viral replication to a dysregulated host immune response [15]. This phase can be characterized by the excessive production of pro-inflammatory cytokines—often referred to as a “cytokine storm”—including interleukin-6 (IL-6), tumor necrosis factor-alpha (TNF-a), and interleukin-1 beta (IL-1b) [16]. The resulting hyperinflammatory state contributes to increased vascular permeability, diffuse alveolar damage, and profound hypoxemia [17]. Accordingly, the therapeutic window is strongly phase-dependent: neutralizing anti-spike mAbs are most effective when administered early during viral replication, whereas host-directed immunomodulatory mAbs are used in the later hyperinflammatory phase (Table 1) [18].

### 2.1. First-Generation Neutralizing mAbs: The Era of Clinical Efficacy

During the dominance of the ancestral Wuhan strain and the subsequent Alpha and Delta variants, first-generation neutralizing monoclonal antibodies (mAbs) showed clinically meaningful efficacy and contributed to reshaping outpatient COVID-19 management [19]. Bamlanivimab, a neutralizing IgG1 mAb derived from convalescent individuals, targets an epitope overlapping the ACE2 binding site [20]. Although early studies suggested benefit, post-authorization experience highlighted the virus’s capacity for rapid escape, and bamlanivimab monotherapy was associated with the selection of resistant variants [21]. To mitigate escape, bamlanivimab was combined with etesevimab, which binds a distinct, non-overlapping RBD epitope [22]. In the BLAZE-1 trial, this dual-antibody strategy reduced viral load and was associated with a lower risk of hospitalization or death in high-risk outpatients [23]. Similarly, the casirivimab–imdevimab cocktail was developed as a high-affinity combination targeting distinct, non-overlapping RBD regions [24], an approach intended to reduce the likelihood of mutational escape [25]. The RECOVERY platform trial supported its benefit not only in outpatients but also in hospitalized patients who were seronegative at admission, with reductions in mortality in this subgroup [26]. In 2021, this cocktail became widely adopted and maintained activity against Delta in clinical use [27]. In addition to these agents, regdanvimab (CT-P59), developed by Celltrion, was evaluated as a first-generation neutralizing anti-spike monoclonal antibody for early outpatient COVID-19. In randomized clinical trials conducted largely prior to Omicron emergence, regdanvimab reduced disease progression in high-risk patients and shortened time to clinical recovery, supporting its use in mild-to-moderate disease during the circulation of earlier variants [28]. As with other first-generation anti-RBD antibodies, its clinical utility was later constrained by reduced susceptibility associated with Omicron-lineage immune escape [29]. While treatment of early infection was the primary focus, immunocompromised individuals (e.g., solid organ transplant recipients and patients with hematologic malignancies) remained at elevated risk because vaccine-induced protection was often suboptimal in these populations. For this reason, tixagevimab–cilgavimab (Evusheld) was developed for pre-exposure prophylaxis. These antibodies incorporated Fc-region modifications (YTE mutations) to extend their half-life beyond six months [30]. In early clinical studies, this strategy reduced the risk of symptomatic COVID-19 over a six-month period, providing a preventive option for highly vulnerable patients [31]. Because neutralizing anti-spike mAbs have limited utility once severe disease is established, immunomodulatory approaches became important in hospitalized patients with systemic inflammation. Tocilizumab, an anti-IL-6 receptor mAb used in inflammatory conditions and cytokine release syndrome, emerged as a key option for critically ill patients [32]. By blocking soluble and membrane-bound IL-6 receptors, tocilizumab attenuates downstream inflammatory signaling implicated in ARDS [33]. The RECOVERY and REMAP-CAP trials showed that tocilizumab, administered alongside systemic corticosteroids (e.g., dexamethasone) in patients with worsening hypoxia and elevated inflammatory markers (such as CRP), improved survival and reduced progression to invasive mechanical ventilation [34,35]. Similar benefits were subsequently reported for sarilumab, another IL-6 receptor antagonist, supporting its use as an alternative, including during drug shortages [36]. Overall, these findings underscore the need for phase-adapted strategies that combine antiviral activity early with host-directed immunomodulation in later stages [37]. The therapeutic landscape changed substantially in November 2021 with the identification of the Omicron (B.1.1.529) variant [38]. Omicron harbored an unusually large number of spike mutations, including multiple substitutions within the RBD (e.g., K417N, T478K, N501Y, and E484A) [39]. Structural and biophysical analyses indicated that these changes altered key antibody epitopes and the antigenic surface of the spike protein [40]. Consistent with these observations, in vitro pseudovirus neutralization assays and subsequent epidemiological data showed markedly reduced susceptibility to several first-generation agents, including bamlanivimab–etesevimab, casirivimab–imdevimab, and later the prophylactic tixagevimab–cilgavimab combination [41,42]. As a result, international regulatory agencies, including the EMA and FDA, revised their recommendations and restricted or withdrew authorizations for multiple products, effectively concluding the first-generation neutralizing mAb era [43].

### 2.2. Later-Generation mAbs: Sotrovimab, Bebtelovimab, and the Evolutionary Arms Race

As first-generation neutralizing antibodies lost activity due to extensive spike mutations, sotrovimab was positioned as a key “bridge” therapy. This monoclonal antibody was derived from a memory B cell isolated from a SARS-CoV-1 survivor of the 2003 outbreak, highlighting the potential of cross-reactive antibody responses. Sotrovimab targets a conserved, non-RBD epitope on the side of the spike protein that is shared across multiple sarbecoviruses [44,45]. Because this region is implicated in the viral fusion process, it is considered less tolerant to mutation than the highly variable receptor-binding domain. Consistent with this rationale, sotrovimab retained neutralizing activity against the Alpha, Beta, Gamma, and Delta variants, and importantly, showed preserved activity against the initial Omicron BA.1 subvariant when several other anti-spike mAbs had reduced effectiveness [46]. In the COMET-ICE trial, early intravenous administration of sotrovimab reduced the risk of disease progression, hospitalization, or death in high-risk patients [47]. However, subsequent Omicron evolution affected susceptibility even for this more conserved target. The emergence of BA.2 and later descendants (e.g., BA.4, BA.5, BQ.1.1, and XBB lineages) was associated with reduced in vitro susceptibility to sotrovimab and higher concentrations required for neutralization [48]. Although Fc-mediated effector functions (e.g., ADCC) may contribute to in vivo activity when neutralization is reduced, clinical deployment of sotrovimab has been substantially restricted by regulatory agencies as variant susceptibility evolved [49]. A similar pattern was observed for bebtelovimab. This antibody was authorized via an accelerated pathway during the BA.2 wave due to strong in vitro neutralization and its targeting of a relatively conserved RBD epitope [50]. However, the rise of BQ.1 and BQ.1.1 subvariants carrying mutations such as K444T and N460K markedly reduced bebtelovimab binding and neutralizing activity, which led to withdrawal of its authorization by late 2022 [51].

## 3. Long COVID and Systemic Complications

The scale of the SARS-CoV-2 pandemic has led to a substantial burden of post-acute sequelae of SARS-CoV-2 infection (PASC), commonly referred to as long COVID. Beyond the acute phase, a proportion of individuals experience persistent, relapsing, or fluctuating symptoms that can last for months and significantly impair quality of life [52,53]. Long COVID is clinically heterogeneous and may involve profound fatigue, post-exertional symptom exacerbation, neurocognitive dysfunction (“brain fog”), dysautonomia (including postural orthostatic tachycardia syndrome, POTS), and cardiovascular manifestations such as persistent chest pain, arrhythmias, or evidence of ongoing endothelial dysfunction [52,53]. These features support the view of COVID-19 as a multisystem disease with potentially prolonged consequences. Although the underlying mechanisms remain incompletely defined, current models suggest a multifactorial pathophysiology. Proposed contributors include persistent viral or antigen reservoirs, chronic endothelial injury with microvascular abnormalities, and sustained immune dysregulation with autoantibody formation and latent virus reactivation [54]. The relative contribution of each pathway likely varies across patient subgroups. These mechanistic hypotheses provide a rationale for investigating whether early antiviral control influences long-term outcomes. By reducing viral load during the initial replication phase, early interventions may theoretically limit systemic dissemination, prolonged antigenic stimulation, and downstream immune perturbations [55]. Within this framework, neutralizing monoclonal antibodies—when effective against circulating variants and administered promptly—may have potential relevance not only for preventing severe acute disease, but also for modulating the risk or severity of post-acute sequelae. However, direct clinical evidence remains limited, and further prospective studies are needed to clarify the relationship between early passive immunotherapy and long COVID outcomes [55,56].

## 4. The Role of Clinical Pharmacy, Antimicrobial Stewardship, and Healthcare Sustainability

The rapid deployment of costly and logistically demanding biological therapies during the COVID-19 pandemic posed substantial operational, economic, and clinical challenges for healthcare systems [57]. Hospital pharmacists played a central role in implementing and governing these therapies, including cold-chain management, aseptic preparation workflows (notably for agents requiring precise, weight-based dilution such as tocilizumab), and adherence to frequently evolving regulatory requirements and national AIFA monitoring registries [58]. Cost-effectiveness remained a key consideration for public health sustainability. As observed with other high-cost biologics such as anti-CGRP monoclonal antibodies in migraine prevention—where economic evaluations and real-world evidence inform appropriate use—the deployment of COVID-19 mAbs required careful pharmacoeconomic oversight to avoid inefficient resource allocation when variant-associated loss of antiviral activity limited clinical value [59]. The pandemic also produced secondary effects on medication use. Psychological distress linked to lockdown measures and infection-related stressors was associated with changes in psychotropic prescribing patterns [60]. In parallel, high hospitalization volumes, diagnostic uncertainty, and concerns about bacterial co-infection contributed to increased use of broad-spectrum antibiotics [61]. Italian retrospective data described a “silent pandemic inside the pandemic”, with spikes in antibiotic consumption potentially accelerating antimicrobial resistance [62]. These findings reinforce the importance of pharmacist-led antimicrobial stewardship and medication safety strategies alongside the implementation of novel antiviral therapeutics [58,63].

## 5. Future Directions and Next-Generation Antibody Therapeutics

**Figure 2:**
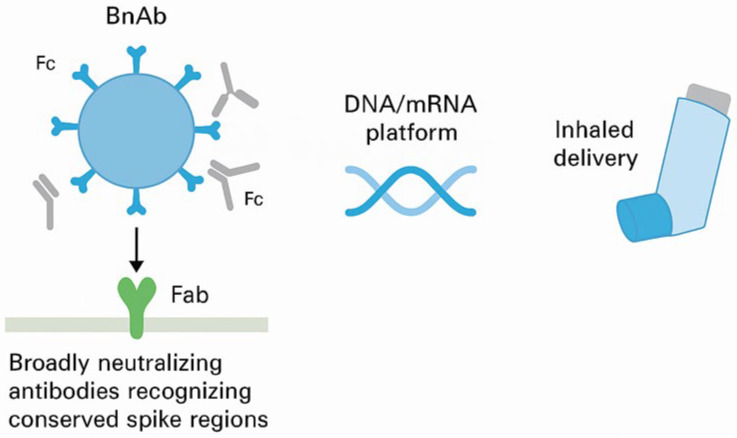
Conceptual overview of next-generation antibody strategies. Broadly neutralizing antibodies (bnAbs) are shown recognizing conserved spike regions via the Fab (variable) domains, while the Fc region is displayed separately to indicate effector functions rather than antigen recognition. The schematic also highlights emerging delivery approaches, including nucleic-acid encoded antibodies (DNA/mRNA platforms) and inhaled administration.

A key lesson from the COVID-19 pandemic is that therapeutically targeting highly mutable, immunodominant regions of RNA viruses may have limited long-term durability. Variant-driven antigenic drift can reduce the clinical usefulness of neutralizing monoclonal antibodies (mAbs) that are optimized for a specific receptor-binding motif, thereby necessitating repeated updates to maintain activity against newly dominant lineages [64]. This experience has emphasized the need to complement variant-matched approaches with strategies that are less sensitive to ongoing spike diversification. Accordingly, current research priorities increasingly include the discovery and engineering of broadly neutralizing antibodies (bnAbs) that target conserved and functionally constrained epitopes. Candidate targets include conserved regions within the receptor-binding domain (RBD) core, and in particular, the S2 fusion machinery. Because the S2 subunit mediates membrane fusion through structurally essential elements (e.g., the fusion peptide and heptad repeat regions), it may be subject to stronger functional constraints than more variable surface-exposed regions [65]. Targeting such conserved sites may support the development of pan-sarbecovirus or potentially broader coronavirus-directed antibody therapies [65]. However, improving the resilience of antibody-based interventions also requires addressing implementation barriers observed during the pandemic. Conventional mAbs often depend on temperature-controlled supply chains and resource-intensive intravenous administration, which can limit timely access and scalability, especially in settings with constrained infrastructure. For this reason, alternative delivery and production platforms are being explored to complement traditional manufacturing and infusion-based deployment. Among these approaches, DNA-encoded monoclonal antibodies (dMAbs) and mRNA-based antibody therapeutics aim to deliver genetic instructions that enable in vivo expression of the desired antibody. By reducing reliance on large-scale protein manufacturing and potentially simplifying distribution requirements, these platforms may help mitigate supply constraints and support longer-lasting prophylaxis in selected high-risk populations (Figure 2) [66]. In parallel, inhaled mAbs are under development to deliver antibodies directly to the respiratory tract, potentially achieving higher local concentrations at the primary sites of viral entry and replication. Such localized administration may enhance early interception at mucosal surfaces and could contribute to reducing transmission, although clinical validation is required [67]. Overall, future preparedness will likely rely on the convergence of structural vaccinology and antibody engineering, data-driven discovery approaches, and delivery platforms that improve accessibility and responsiveness to viral evolution [64,65,66,67].

## 6. Conclusions

Monoclonal antibodies have undeniably been indispensable, life-saving tools in mitigating the devastating morbidity and mortality of the COVID-19 pandemic. First-generation therapies shielded millions before being outmaneuvered by the hyper-mutated Omicron lineage. Immunomodulators like tocilizumab remain vital, immovable pillars for managing severe systemic inflammation in the ICU. The complex trajectory of agents like sotrovimab highlights both the incredible ingenuity of targeting conserved viral epitopes and the relentless, Darwinian adaptability of SARS-CoV-2. Moving forward into the endemic phase, the integration of variant-proof therapeutics, real-time genomic surveillance, and robust, pharmacist-led healthcare sustainability models will be absolutely essential to manage the long-term reality of COVID-19 and prepare our healthcare infrastructures for future viral threats.

## Figures and Tables

**Table 1:** Classification and chronology of monoclonal antibodies against COVID-19.

Monoclonal Antibody	Category/Generation	Target/Mechanism of Action	Reference Clinical Trials	Efficacy and Impact of Variants (Especially Omicron)
Bamlanivimab (monotherapy)	Neutralizing (First Generation)	Binds an RBD epitope overlapping the ACE2 binding site.	Early clinical studies/initial authorization experience.	Monotherapy was associated with emergence of resistant escape variants; single-agent use was discontinued.
Bamlanivimab + Etesevimab	Neutralizing cocktail (First Generation)	Two antibodies binding distinct, non-overlapping RBD epitopes.	BLAZE-1 (reduced hospitalization/death in high-risk outpatients).	Active against Alpha/Delta; markedly reduced neutralization with Omicron, limiting clinical utility.
Casirivimab + Imdevimab	Neutralizing cocktail (First Generation)	Two antibodies targeting distinct RBD regions to reduce escape.	RECOVERY (benefit in seronegative hospitalized patients; reduced mortality in subgroup).	Widely used in 2021 and effective against Delta; substantially reduced activity with Omicron.
Regdanvimab (CT-P59)	Neutralizing (First Generation)	Anti-RBD mAb that blocks spike–ACE2 interaction.	Phase 2/3 and Phase 3 randomized trials (NCT04602000).	Demonstrated clinical benefit in pre-Omicron settings; reduced susceptibility with Omicron and later lineages constrained use.
Tixagevimab + Cilgavimab (Evusheld)	Pre-exposure prophylaxis (Long-acting)	Fc-modified antibodies (YTE mutations) to extend half-life (>6 months).	Early prophylaxis trials (reduced symptomatic COVID-19 risk over 6 months).	Provided protection for immunocompromised patients; reduced activity with Omicron sub-lineages limited effectiveness and use.
Tocilizumab	Immunomodulator (Severe Phase)	IL-6 receptor antagonist (soluble and membrane-bound), reduces inflammatory signaling.	RECOVERY, REMAP-CAP (improved survival; reduced progression to mechanical ventilation when used with corticosteroids).	Host-directed mechanism; not affected by spike mutations; remains clinically relevant in selected severe cases.
Sarilumab	Immunomodulator (Severe Phase)	IL-6 receptor antagonist (similar to tocilizumab).	Clinical studies/observational evidence (often used as alternative).	Used as an alternative option, including during tocilizumab shortages; host-directed mechanism.
Sotrovimab	Neutralizing (Later Generation)	Targets a more conserved non-RBD epitope; activity may include Fc effector contribution.	COMET-ICE (reduced progression risk in high-risk outpatients).	Active against several pre-Omicron variants and early Omicron BA.1; reduced susceptibility to BA.2/BA.4/BA.5/XBB led to restricted deployment.

## Data Availability

No new data were created or analyzed in this study. Data sharing is not applicable to this article.

## Abbreviations

ACE2	Angiotensin-Converting Enzyme 2
ADCC	Antibody-Dependent Cellular Cytotoxicity
ADCP	Antibody-Dependent Cellular Phagocytosis
AIFA	Italian Medicines Agency (Agenzia Italiana del Farmaco)
ARDS	Acute Respiratory Distress Syndrome
bnAbs	Broadly Neutralizing Antibodies
CDC	Complement-Dependent Cytotoxicity
CGRP	Calcitonin Gene-Related Peptide
COVID-19	Coronavirus Disease 2019
CRP	C-Reactive Protein
dMAbs	DNA-Encoded Monoclonal Antibodies
DNA	Deoxyribonucleic Acid
EMA	European Medicines Agency
Fc	Fragment Crystallizable
FcγRs	Fc Gamma Receptors
FDA	Food and Drug Administration
ICU	Intensive Care Unit
IgG1	Immunoglobulin G Subclass 1
IL-1b	Interleukin-1 Beta
IL-6	Interleukin-6
LNP	Lipid Nanoparticle
mAbs	Monoclonal Antibodies
MALT	Mucosal-Associated Lymphoid Tissue
ME/CFS	Myalgic Encephalomyelitis/Chronic Fatigue Syndrome
mRNA	Messenger Ribonucleic Acid
PASC	Post-Acute Sequelae of SARS-CoV-2 infection
POTS	Postural Orthostatic Tachycardia Syndrome
RBD	Receptor-Binding Domain
RNA	Ribonucleic Acid
S protein	Spike Protein
SARS-CoV-2	Severe Acute Respiratory Syndrome Coronavirus 2
TMPRSS2	Transmembrane Serine Protease 2
TNF-a	Tumor Necrosis Factor-alpha
VOCs	Variants of Concern
